# In Vivo Evidence for Voltage-Gated Sodium Channel Expression in Carcinomas and Potentiation of Metastasis

**DOI:** 10.3390/cancers11111675

**Published:** 2019-10-28

**Authors:** Mustafa B. A. Djamgoz, Scott P. Fraser, William J. Brackenbury

**Affiliations:** 1 Department of Life Sciences, Neuroscience Solutions to Cancer Research Group, Imperial College London, South Kensington Campus, London SW7 2AZ, UK; s.p.fraser@imperial.ac.uk; 2 Department of Biology and York Biomedical Research Institute, University of York, Heslington, York, YO10 5DD, UK; william.brackenbury@york.ac.uk

**Keywords:** metastasis, voltage-gated sodium channel, embryonic splice, imaging, electrolytes, xenografts, epidemiology

## Abstract

A wide body of evidence suggests that voltage-gated sodium channels (VGSCs) are expressed de novo in several human carcinomas where channel activity promotes a variety of cellular behaviours integral to the metastatic cascade. These include directional motility (including galvanotaxis), pH balance, extracellular proteolysis, and invasion. Contrary to the substantial in vitro data, however, evidence for VGSC involvement in the cancer process in vivo is limited. Here, we critically assess, for the first time, the available in vivo evidence, hierarchically from mRNA level to emerging clinical aspects, including protein-level studies, electrolyte content, animal tests, and clinical imaging. The evidence strongly suggests that different VGSC subtypes (mainly Nav1.5 and Nav1.7) are expressed de novo in human carcinoma tissues and generally parallel the situation in vitro. Consistent with this, tissue electrolyte (sodium) levels, quantified by clinical imaging, are significantly higher in cancer vs. matched non-cancer tissues. These are early events in the acquisition of metastatic potential by the cancer cells. Taken together, the multi-faceted evidence suggests that the VGSC expression has clinical (diagnostic and therapeutic) potential as a prognostic marker, as well as an anti-metastatic target. The distinct advantages offered by the VGSC include especially (1) its embryonic nature, demonstrated most clearly for the predominant neonatal Nav1.5 expression in breast and colon cancer, and (2) the specifically druggable persistent current that VGSCs develop under hypoxic conditions, as in growing tumours, which promotes invasiveness and metastasis.

## 1. Introduction

Cancer is a major killer in the modern world with one in two men and one in three women expected to be diagnosed with some kind of cancer during their lifetime. Some 9.6 million cancer-related deaths were estimated globally in 2018 [1,2]. The current statistics are expected to rise by more than 70% over the next two decades, reaching 17 million annual deaths by 2030 [1]. Causes include longevity, as well as unhealthy diets and lifestyles, leading to obesity and type-2 diabetes, which are interrelated and promote cancer incidence. Although great progress has been made in clinical management of cancer, many problems nonetheless remain. These include uncertainties in diagnosis, limited effectiveness, and undesirable side effects of major treatment modalities such as chemotherapy, radiotherapy, and biological therapies. Furthermore, cancer treatment, especially novel therapies such as immunotherapy, can be very costly. In conclusion, there is a great unmet need in cancer management. In this regard, ion channels have emerged in the last two decades as promising new targets [3,4,5,6]. It is likely that cancer cells/tissues express as many types of ion channels as the brain, the classic home of ion channels, and these contribute to different components and stages of the dynamic process of cancer development and progression, as well as response to therapy. 

We have placed particular emphasis on voltage-gated ion channels (VGICs) due to the profound impact upon cellular processes of membrane potentials (equivalent to some 10,000,000 V/m). With regard to metastasis, the main cause of death from cancer, a substantial body of evidence suggests voltage-gated sodium channels (VGSCs) are expressed de novo in cells derived from a wide range of carcinomas. Specifically, invasive cancer cells with strong metastatic ability have been shown to express functional VGSCs. The initial discovery was made in rat prostate cancer cells and then confirmed in humans [7,8]. Subsequently, several other carcinoma cell lines were also shown to express functional VGSC(s). These include cancers of breast, prostate, colon, lung, stomach, cervix, ovary, and skin (see Table 1). Interestingly, of the nine functional subtypes of VGSC, in these carcinomas, two appeared as the most prevalent—Nav1.5 and Nav1.7 (Table 1). 

Importantly, where studied, the VGSC upregulation was found to be accompanied by downregulation of outward (mainly K^+^) currents [7,8,9,20,31]. Such combination of VGICs would make membranes of strongly metastatic cancer cells potentially electrically ‘excitable’. This was formulated as the “Celex Hypothesis”, proposing that it is this membrane excitability that makes metastatic cells aggressive and hyperactive, leading to disruption of their surroundings [32,33]. Indeed, a recent study has shown that the strongly metastatic human prostate cancer PC-3 cells undergo spontaneous regenerative spike activity, although these were thought to be driven by Ca^2+^ [34]. A further study extended this approach to ex vivo recording of electrical activity (local field potentials) associated with breast cancer cells implanted in the cortex of mice. Thus, it was shown that aberrant spontaneously-occurring spike events occurred within the intact tumour microenvironment [35].

As regards mechanisms, VGSCs can function in two distinct modes—conducting and non-conducting. In turn, the conducting mode can generate two kinetically distinct currents—transient (I_NaT_) and persistent (I_NaP_). The latter is promoted by hypoxia, well known to occur in growing tumours, and may be responsible for elevating the level of Na^+^ in cancer tissues (see the section on ‘tissue electrolytes’). Indeed, I_NaP_ has been proposed as a major anti-metastatic target [13,36]. The non-conducting (‘non-canonical’) mode may involve the alpha or the beta subunit through protein–protein interactions and immunoglobulin-like cell adhesion effects, respectively. The latter may be mediated by beta subunits, which may also be expressed independently of alpha subunits. Regarding the alpha subunit, most evidence points to the sodium-hydrogen exchanger 1 (NHE1) as an interacting/downstream partner. Thus, VGSC-activated NHE1 can acidify the pericellular space, leading to extracellular proteolysis and promotion of invasiveness [37]. Subsequently, Src kinase and invadopodial activity were also demonstrated to be involved in the invasiveness [38].

In spite of the substantial in vitro evidence for functional VGSC expression in invasive cancer cells and the emerging mechanistic insights, however, work to determine in vivo relevance of the findings has been relatively sporadic [39]. Here, we assess, for the first time in depth, the available in vivo evidence hierarchically from tissue mRNA expression to whole-animal and clinical studies. The main emphasis is on carcinomas; haematological and brain cancers are beyond the scope of this review because their pathophysiology may be different.

## 2. mRNA Level Studies

Multiple VGSCs have been detected at mRNA level for individual human cancers. Fraser et al. (2005) described Nav1.5–1.7 transcripts in human breast cancer (BCa) biopsy tissues [9]. On the other hand, prostate cancer (PCa) biopsies were found to express Nav1.2/1.3/1.5–1.7/1.9 mRNAs [16]. Nav1.7 was dominant, consistent with VGSC currents recorded from strongly metastatic PC-3 cells being tetrodotoxin-sensitive (TTX-S) [8]. Human malignant pleural mesothelioma cells in primary culture expressed Nav1.6 and Nav1.7 mRNAs most consistently [20]. Human malignant cervical cancer cells from smear biopsies in primary culture expressed Nav1.2/1.4/1.6/1.7 mRNAs, with Nav1.6 being dominant [21,22]. 

**Figure 1:**
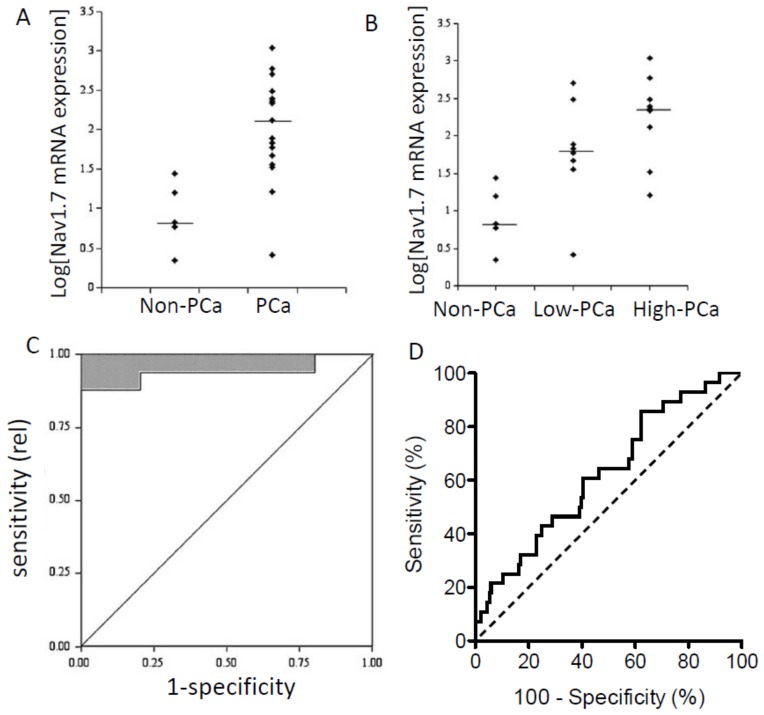
VGSC mRNA expression and associated ‘receiver operator characteristics’ of human prostate and breast cancers. (**A**–**C**) Data for human prostate cancer (PCa). Nav1.7 mRNA levels in non-PCa vs. PCa patient samples determined by real-time PCR. “Non-PCa” were mostly benign prostatic hyperplasia. Logarithmic plots of Nav1.7 expression levels normalized to beta-actin are shown. (**A**) Non-PCa vs. PCa. (**B**) Comparisons of non-PCa, low-grade PCa (Gleason grade < 6), and higher grade PCa (Gleason grade > 6). Medians are shown as short horizontal lines in both (**A**) and (**B**). (**C**) “Receiver operator characteristics” (ROC) analysis showing the potential diagnostic efficacy of Nav1.7 mRNA expression in distinguishing non-PCa (*n* = 5) from PCa (*n* = 17). Efficacy is indicated by upper-left deviation from the non-discriminatory diagonal line. (**D**) ROC analysis as in (**C**) but for breast cancer (*n* = 181). Modified from [16] (**A**–**C**), and [41] (**D**).

Importantly, VGSC mRNA expression has been shown to correlate with disease progression in several carcinomas and, thus, may have prognostic implications. In human prostate biopsies the Nav1.7 mRNA level was significantly higher in (i) cancer vs. non-cancer (mainly ‘benign prostatic hyperplasia’/BPH) tissues and (ii) high-grade (Gleason score > 6) vs. low-grade tumours (Figure 1A,B) [16]. Receiver operating characteristic (ROC) analysis revealed that the Nav1.7 mRNA expression had sufficient specificity and selectivity to be considered a viable diagnostic biomarker for PCa (Figure 1C) [16]. In a later study, expression of Nav1.1–1.3, Nav1.5–1.7, and Nav1.9 mRNAs were found in normal prostate and Nav1.1–1.7 for BPH. Importantly, however, transcript levels of Nav1.6 and Nav1.7, respectively, were 6- and 27-fold higher in PCa compared with normal or BPH samples [40]. 

In conclusion, most evidence suggests (i) that Nav1.7 is the dominant VGSC mRNA species in human PCa and (ii) that the expression/upregulation has diagnostic/prognostic potential.

For human BCa, initial comparative PCR studies on strongly metastatic (MDA-MB-231) cells with weakly/non-metastatic (MCF-7) cells revealed Nav1.5 mRNA to be (ca. 1800-fold) higher, consistent with the VGSC current in the former being TTX-resistant (TTX-R) [9]. A double-blind test on 20 patients revealed that the expression of Nav1.5 mRNA in breast biopsies was significantly directly correlated with the presence of metastasis in lymph nodes (LNMs) in ~75% of cases. There was no case of LNM without Nav1.5 expression. The remaining ~25% were Nav1.5-positive but LNM-negative, raising the possibility that the Nav1.5 expression in breast tissue had occurred but metastases had not yet developed, that is, that Nav1.5 expression is an early event in the acquisition of metastatic potential [9]. Consistent with this, an in silico study on colon cancer concluded that *SCN5A* (the gene encoding Nav1.5) expression was upstream of several canonical invasiveness-associated genes, including those for Ca^2+^ signalling, Wnt signalling, mitogen-activated protein (MAP) kinase, proteases, and membrane remodelling/secretion [14]. 

**Figure 2:**
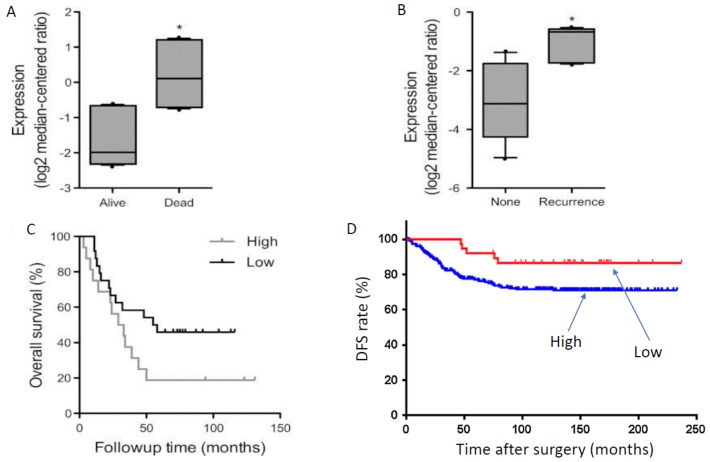
Positive associations between VGSC (Nav1.5) expression and clinical behaviour of breast and colon cancers. The channel is presumed to be ‘neonatal’ Nav1.5. (**A**) Patients who died of breast cancer expressed significantly more Nav1.5 mRNA than those who were alive. * = *p* < 0.05. (**B**) Breast cancer patients whose cancer recurred expressed significantly more Nav1.5 mRNA than those who were still cancer-free. * = *p* < 0.05. (**C**) Overall survival was significantly longer for breast cancer patients expressing low levels of Nav1.5 mRNA, compared with those with high levels of expression (*n* = 181). (**D**) Disease-free survival (DFS) of colon cancer patients in relation to Nav1.5 protein expression (*n* = 23 and 183 for low- and high-expression, respectively; *p* = 0.032). Time is for months after radical resection. Modified from [41] (**A**–**C**) and [42] (**D**).

A further study also showed Nav1.5 mRNA to be significantly (3.6-fold) higher in invasive BCa compared to normal breast tissue [41]. Importantly, Nav1.5 mRNA expression was also significantly higher in patients (i) who died rather than survived the disease (Figure 2A), (ii) with disease recurrence vs. non-recurrence (Figure 2B), and (iii) whose survival was poorer (Figure 2C) [41]. As in the case of PCa, ROC analysis indicated Nav1.5 mRNA expression in human BCa to have sufficient specificity and selectivity to be considered a viable diagnostic biomarker (Figure 1D) [41]. 

In human cervical cancer biopsies, Nav1.6 mRNA levels were ~40-fold higher than in non-cancerous cervical tissues [22]. Similarly, the mRNA expression of a Nav1.7 splice-variant was ~20-fold higher in cancer than normal tissue [22]. Interestingly, several different Nav1.6 mRNA splice variants (from Exon 18) that would encode non-functional protein were also identified in cervical tissue biopsies [43]. However, the variant 18A encoded a functional protein and its expression correlated with cancer progression, being detected in only 58% of non-cancerous tissues, but 75% of neoplasia, and 100% of cervical cancer samples positive for human papilloma virus type 16. Subsequent work thus focused on Nav1.6 as the VGSC driving the invasiveness [43]. 

Although VGSC expression has not been studied in colorectal carcinoma (CRCa) tissues in detail at the mRNA level, cell-based studies suggested Nav1.5 expression to be predominant, consistent with the TTX-R nature of the VGSC currents [14,15]. Importantly, using three different siRNAs, Guzel et al. (2019) showed that Nav1.5 (specifically the neonatal splice variant, nNav1.5) was primarily responsible for the VGSC-dependent component of invasiveness [13]. Interestingly, in CRCa, a significantly lower level of Nav1.6 mRNA expression was found in tumour tissues compared with paired neighbouring non-cancerous tissues [44]. This difference was more pronounced for patients below the age of 45 and correlated significantly with gender, tumour grade, location, and histopathological classification [44]. At present, it is not clear if the Nav1.6 mRNA expression is translated to protein, especially as the VGSC currents are TTX-R, which would rule out Nav1.6 being functional. In conclusion, evidence favours nNav1.5 as being primarily responsible for promoting the metastatic process in CRCa. 

In human gastric cancer biopsy tissues, a detailed study by Xia et al. (2016) showed via real-time-PCR analyses that Nav1.7 (*SCN9A*) was the most highly expressed VGSC subtype [23]. An earlier study also found functional Nav1.5 expression in vitro, but this was not confirmed in vivo [45]. Finally, for human ovarian cancer, Nav1.1, 1.3–1.5, mRNA levels were significantly higher (ca. 10-fold for Nav1.5) in cancer cells compared with benign ovarian tumour or normal ovary [24]. It was Nav1.5 mRNA expression, however, that was proposed to be increased at the transition from normal to benign and then to cancer, and was even higher in ovarian cancers with lymph node metastases than those without [24]. This brings up the possibility of an in vivo–in vitro mismatch, as Nav1.5 mRNA (which, if functionally expressed) would give rise to a TTX-R channel, whereas the predominant functional VGSC in the SKOV-3 ovarian cancer cell line is TTX-S [46]. 

Importantly, where studied, the predominant VGSC has been shown to be consistent with a developmentally regulated ‘neonatal’ splice form. This is most pronounced in Nav1.5, wherein the switch from the 3’ (‘adult’) to the 5’ (‘neonatal’) form of exon 6 generates a difference of 31 nucleotides [9]. This results in a change in seven amino acids in the extracellular loop of segments 3–4 in domain 1 [9]. In particular, the amino acid at position 211 switches from a negatively charged aspartate in ‘adult’ to a positively charged lysine in ‘neonatal’, that is, a double charge change. Similar splicing for Nav1.6 and Nav1.7 would result in the ‘homologous’ aspartate switching to an arginine. Predominant nNav1.5 expression has been demonstrated most extensively in BCa, including in human biopsies [9,10]. It also occurs in colon cancer, and possibly melanoma, astrocytoma, neuroblastoma, and ovarian cancer (Table 1). This ‘oncofoetal’ phenomenon is consistent with the dedifferentiated nature of cancer tissues. 

From the available mRNA-level evidence, three main conclusions can be drawn. First, expression of VGSC mRNA(s) is significantly higher in cancer relative to comparable normal/non-cancerous tissues. Second, multiple VGSC mRNA transcripts occur in human cancers. Although one of these appears to be dominant in one condition, it is possible that dynamic changes occur during the progression of the disease and/or in response to therapy. Third, importantly, where studied, the mRNA expression has been found to be in ‘neonatal’ splice forms. This aspect has significant implications for the clinical applicability of VGSCs. Nevertheless, the presence of mRNA(s) does not guarantee protein expression (e.g., [47,48,49]). The in vivo evidence for VGSC protein expression in cancers is discussed in the following section.

## 3. Protein Expression

VGSC protein expression has been shown to occur in several cancers and correlates generally with disease progression/metastatic potential. Functional VGSC protein has been detected ex vivo. Primary cultures of cervical cancer epithelial cells were shown to express TTX-S VGSC currents [21]. Further work using Cn2, a Nav1.6-specific toxin, revealed activity of Nav1.6 channels in the plasma membrane of these cells [22]. Consistent with this, robust Nav1.6 immunoreactivity was detected in cervical cancer biopsies and primary cultures at levels much higher than normal cervical tissues, with expression widely distributed in both cytoplasm and plasma membrane [22]. Importantly, both TTX (6 μM) and Cn2 (1 μM) reduced invasiveness of the primary cervical cancer cells by ~20% [22]. Interestingly, immunohistochemistry also revealed Nav1.7 protein expression in cervical cancer biopsies, but its possible functional relevance was not studied. Similarly, a TTX-S VGSC current (IC_50_ = 16 nM) was detected in primary cultures of human malignant pleural mesothelioma cells, but not in normal mesothelial cells [20]. As in all other carcinomas studied, channel activity significantly enhanced the cells’ motility [20]. Interestingly, in agreement with the Celex Hypothesis, the VGSC upregulation (de novo expression) was accompanied by a significant reduction in the amplitude of ‘outward’ voltage-gated K^+^ current [20]. 

**Figure 3:**
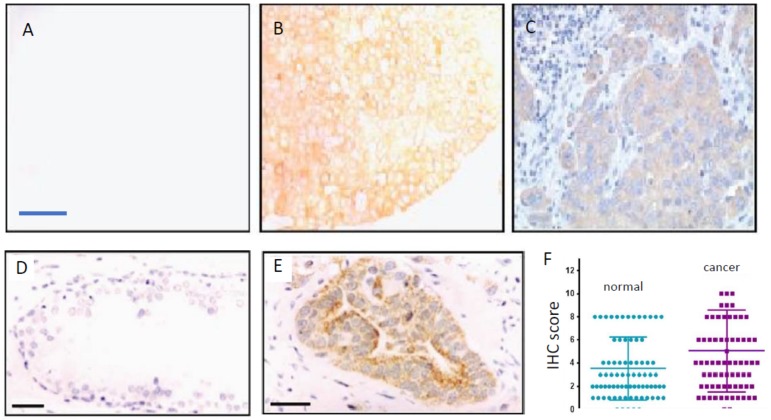
VGSC protein expression in breast, prostate, and colon cancers. VGSC alpha-subunit protein expression in human cancer biopsy tissue sections was determined by immunohistochemistry (IHC). (**A**–**C**) Typical immunostaining of human breast tissue with nNav1.5-specific polyclonal antibody (NESOpAb). Little staining was detected in normal human breast tissue, as illustrated in (**A**), whereas strong heterogeneous staining was detected in the corresponding image from breast cancer (**B**). Sections were also counterstained with haemotoxylin to confirm tissue integrity (**C**). Scale bar, shown in (**A**) is 50 μm and is applicable also to (**B**) and (**C**). (**D**,**E**) Typical staining of human prostate tissue sections exhibiting morphological features of low-grade prostatic intraepithelial neoplasia (PIN) (**D**), and high-grade PIN (**E**). A pan-VGSC alpha-subunit antibody was used. Sections were also counterstained with haemotoxylin to confirm tissue integrity. Scale bar, 50 μm is applicable to (**D**) and (**E**). (**F**) Quantitative data showing significantly higher level of Nav1.5 IHC staining of colon cancer compared with matched normal tissue (*p* < 0.001; *n* = 78). Modified from [9] (**A**–**C**); [16] (**D**,**E**); and [42] (**F**).

In human prostate biopsies and tissue microarrays, VGSC protein expression was found to be significantly higher in cancer vs. non-cancer (Figure 3D,E) [16,50]. A pan-VGSC antibody was used in these studies due to the unreliability of available antibodies for Nav1.7, the predominant VGSC subtype in PCa. However, no correlation was found between the intensity of the VGSC immunoreactivity and Gleason score (GS), as well as the level of prostate-specific antigen [50]. Expression was apparent in PCa cases with a GS of 7, often taken to be the transition to malignancy [51]. This is consistent, again, with VGSC upregulation being an early marker of metastatic progression, as has been suggested also for BCa [9] and colon cancer [14]. Interestingly, statistically significant association was found between Nav1.8 protein expression in human PCa and pathological stage, GS, and lymph node involvement [52]. However, the quality of the immunohistochemistry in this study is not clear because unexpected nuclear staining was apparent. Furthermore, possible in vivo–in vitro mismatch aside, Nav1.8 is a TTX-R VGSC, whereas human PCa cells express TTX-S channels (mainly Nav1.7), and anti-invasive/metastatic effects can be obtained with sub-micromolar concentrations of TTX [8,53].

In human ovarian cancer, western blot assays suggested that there was almost no Nav1.5 protein expression in normal ovary, but this was higher in cancer biopsy tissues [24]. Immunohistochemistry also revealed the expression of Nav1.5 protein in epithelial cells of ovarian cancer biopsies tissues to be much higher than normal ovary [24]. However, again, possible in vivo–in vitro mismatch aside, the suggested ‘predominant’ expression of Nav1.5 (a TTX-R channel) in ovarian cancer is at odds with the finding of a functional TTX-S channel in the SKOV-3 ovarian cancer cell line [46]. In summary, whilst it would appear that upregulated VGSC protein expression occurs in human ovarian cancer, more work is needed to elucidate the nature of the VGSC mRNA(s) and protein(s) expressed. 

For small-cell lung carcinoma, an immunohistochemical study demonstrated VGSC protein upregulation in tumour tissue [54]. A later, more detailed study, which identified Nav1.7 mRNA as the dominant VGSC subtype in several non-small cell lung carcinoma (NSCLC) cell lines, confirmed that Nav1.7 protein expression was also markedly higher for tumour vs. normal lung tissue [19]. It was considered possible that increased Nav1.7 protein expression accompanied the transition from low- to high-grade tumours [19]. This study also demonstrated, albeit in vitro, that the pro-invasive effect of epidermal growth factor (EGF) was mediated largely through the VGSC (Nav1.7). If this observation can (i) be validated in an in vivo animal model and (ii) be demonstrated in human biopsies, for example, in the form of VGSC + EGF/EGF receptor (EGFR) co-localization, it could form the basis of a novel therapy of NSCLC, for example, combining an EGFR tyrosine kinase inhibitor with a VGSC blocker.

As described in Section 2 above, the alternative splicing of exon 6 results in the functional expression of a neonatal form of Nav1.5 in BCa cells [9,10]. The aspartate-to-lysine switch was shown to be responsible for key electrophysiological differences between the two splice variants and, thus, is likely to give rise to pharmacological differences that may be used therapeutically [55]. Importantly, the amino acid differences enabled the production and validation of a polyclonal antibody (“NESOpAb”) with greater than two orders of magnitude blocking selectivity for nNav1.5 over ‘adult’ Nav1.5 [56].

In human BCa biopsy sections, the use of NESOpAb demonstrated that nNav1.5 protein expression was markedly upregulated, compared with normal breast tissues (Figure 3A–C) [9]. Densitometric analysis confirmed the difference to be highly significant as regards both the intensity and the extent of the staining [57]. The latter study also confirmed the lack of expression of nNav1.5 protein expression in a range of other normal human tissues [57]. Thus, nNav1.5 protein expression appears to be cancer-specific [57]. A separate study using an antibody that recognises both nNav1.5 and adult Nav1.5 showed a similar significant up-regulation in breast tumours compared with surrounding normal tissue [11].

In colon cancer, Nav1.5 protein expression has been demonstrated to be at levels significantly higher than matched normal tissues (Figure 3F) [14,42]. However, in line with the in vitro data, the predominant VGSC is considered to be nNav1.5 [13,58]. Indeed, a preliminary study using the NESOpAb antibody demonstrated significantly higher nNav1.5 protein levels for colon cancer biopsy tissues compared with matched mucosa (normal tissues) [58]. An early study had reported expression of a VGSC (unknown subtype) in normal colonic epithelial using a pan-VGSC antibody, but no comparison with cancer was made [59]. Expression was mostly restricted to the cytoplasm or luminal surfaces of the epithelial cells—the subcellular distribution implying that intracellular trafficking of functional protein may occur under appropriate conditions [60]. Importantly, as in the case of BCa, the Nav1.5 expression level correlated with unfavourable disease-free survival (Figure 2D) [42]. Also, high-level Nav1.5 expression was associated positively with estrogen receptor-β expression [42], implying that VGSC/nNav1.5 expression in colon cancer may be under estrogenic control, as in BCa [61] and some neurones [62,63]. As already suggested for the EGF connection in NSCLC, this could form the basis of further combination therapy options for metastatic colon cancer. 

In conclusion, VGSC protein expression in cancer tissues generally but not universally reflects the in vitro cellular studies. We should note, however, that publicly available databases, such as the Human Protein Atlas, or even the Cancer Proteome Atlas, have limited, even conflicting, information [50]. Such apparent discrepancies, in case of the latter, may be due to the quality of the available VGSC ‘subtype-specific’ antibodies. On the other hand, studies of cancers expressing nNav1.5 have given more consistent data thanks to the availability of the highly specific NESOpAb antibody. 

## 4. Tissue Electrolytes

**Figure 4:**
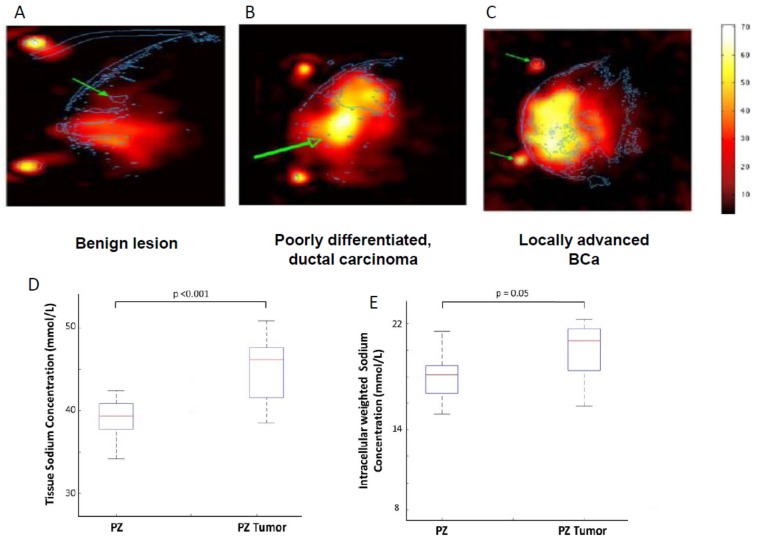
Clinical measurement of tissue sodium levels in breast cancer (BCa) and prostate cancer (PCa) with magnetic resonance imagining (MRI). (**A**–**C**) ^23^Na-MRI’s from three different BCa patients. (**A**) A benign lesion (proliferative fibrocystosis), indicated by the arrow, aligned from gadolinium-enhanced image. (**B**) Infiltrating poorly differentiated ductal carcinoma (outlined in blue). Below (indicated by the green arrow) is a region with edema. (**C**) A large locally advanced BCa (outline in blue). The arrows indicate positioning landmarks. The intensity scale on the far right indicates approximate sodium concentrations (relative). Modified from [66]. (**D**,**E**) Magnetic resonance spectroscopic imaging (MRSI) of prostate. (**D**) Box plots of the tissue sodium concentration in normal and PCa tissue in peripheral zone (PZ) showing significantly increased sodium level in the latter. (**E**) Same as (**D**), but showing intracellular-weighted sodium concentration. Modified from [76].

Currently, there is no report of VGSC activity recording from intact cancers in human patients. However, as already noted, it has been demonstrated that VGSC currents can be recorded from primary cultures of human cervical cancer and mesothelioma cells taken from biopsies [20,21]. Also, Nelson et al. (2015) showed that VGSC activity can be recorded in slices of BCa tumour taken from mouse xenografts [11]. Thus, it would appear that functional VGSC expression is maintained in cancer cells isolated from tumours and tissues. VGSC activity would implicitly lead to a rise in the concentration of intracellular Na^+^, and this has been measured in vitro [31,64]. Such a rise could be detectable in vivo as an increase in tissue Na^+^ level [65]. Indeed, Ouwerkerk et al. (2007) showed by ‘non-invasive’ semi-quantitative ^23^Na–magnetic resonance imaging (MRI) that the Na^+^ content of locally advanced BCa tissue was higher than poorly differentiated ductal carcinoma which, in turn, was higher than benign tissue (Figure 4A–C) [66]. A comparable result was obtained using ^23^Na-MRI for PCa, wherein tissue Na^+^ levels correlated with Gleason grading [67]. This was also observed for ovarian cancer [68]. In a rat model of hepatocellular carcinoma, it was also concluded that tumours had increased levels of tissue Na^+^ and intracellular Na^+^ [69]. Importantly, as well as being an early indicator of metastatic disease, the Na^+^ content of tumours (as measured by ^23^Na-MRI) has also been suggested as being useful in monitoring the effectiveness of therapy [70,71,72,73,74]. Another measurement technique, complementary to ^23^Na-MRI, is magnetic resonance spectroscopic imaging (MRSI), which uses radio frequency transmission reception to map relative concentrations of metabolic markers of disease [75]. Thus, using MRSI, Barrett et al. showed that PCa tissue Na^+^ levels were significantly higher than those in adjacent normal tissue (Figure 4D,E) [76].

Electrolyte analyses have also been carried out on human body fluids. Thus, Sisman et al. (2009) showed that extracellular fluid from breast cysts associated with higher risk of developing cancer had lower Na^+^, higher K^+^, and, hence, a lower Na^+^:K^+^ concentration ratio than those with lower risk [77]. These changes would appear to mirror those seen in tissue. It is well known that cancer is associated clinically with various changes in body electrolyte levels [78]. One of these is “hyponatraemia”, where serum sodium level is reduced in parallel with a poor prognosis [79]. However, it is not currently known if this involves tumour VGSC activity. Such assessments of tumour-associated changes in body electrolyte levels (“fluid cytology”) would offer the advantage of being ‘low-tech’, hence cheaper and potentially more readily available to pathologists. 

Importantly, the Na^+^ influx expected from VGSC-driven (millisecond long) action potential activity would raise intracellular Na^+^ only in the picomolar range [80]. Thus, this is an unlikely source of the increased Na^+^ levels of cancer tissues and unlikely to make an impact on Na^+^-dependent metastatic cell behaviours. A more likely mechanism is the persistent current component of the VGSC. This is enhanced under hypoxic conditions that develop naturally in growing tumours. I_NaP_ can outlast the transient current by hundreds of milliseconds–seconds and could well lead to millimolar changes in the intracellular/tissue level of Na^+^, sufficient to significantly affect Na^+^-dependent cellular mechanisms including NHE1 activity. Indeed, inhibiting I_NaP_ (e.g., by ranolazine) has been shown to produce anti-invasive effects on colon cancer and BCa in vitro [13,81]. Also, as detailed in the next section, ranolazine has been shown to inhibit BCa and PCa metastasis in vivo [12,50]. Accordingly, I_NaP_ inhibitors may serve clinically as anti-metastatic drugs [36]. 

## 5. In Vivo Animal Tests

Several preclinical animal studies have been carried out to evaluate the involvement of VGSCs in tumour progression in vivo. In the first such study, a subcutaneous allograft model of PCa using metastatic Mat-LyLu cells injected subcutaneously in the Copenhagen rat was used [53]. TTX (200 nM; every 2 days) was injected directly into the primary tumour (i) to ensure access to the metastasising cells, (ii) to eliminate any possible side effect because TTX is a highly specific blocker of VGSCs, and (iii) to avoid systemic toxicity because the toxin is potentially lethal. This treatment regime significantly reduced the number of lung metastases at a level greater than 40%. Presumably as a result of the reduction in the metastatic burden, there was also a significant increase in survival by some 20% [53]. Batcioglu et al. (2012) adopted the rat DMBA (7,12-dimethylbenz(a)anthracene) model of BCa with systemic application of RS100642 [82], an analogue of mexiletine, a blocker of TTX-R VGSCs, including Nav1.5 [83]. This study showed that a range of antioxidant responses (including superoxide dismutase, glutathione peroxidase, malondialdehyde) were suppressed significantly, presumably by reduction of the tumour burden. Importantly, also, as in the study of Yildirim et al. (2012) [53], survival was increased by some 26% [82]. These early data suggested that VGSC inhibition may have therapeutic value in vivo.

**Figure 5:**
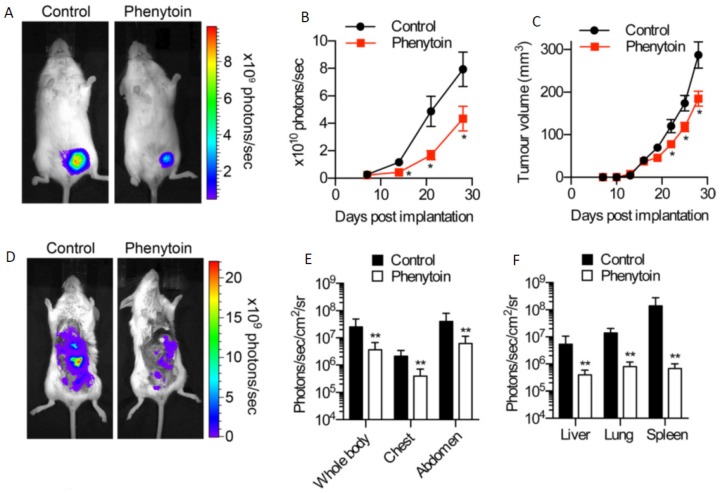
In vivo evidence showing suppression of breast cancer metastasis in a mouse (‘orthotopic’) model by blocking VGSC activity with phenytoin. (**A**) Bioluminescent images of control and phenytoin-treated mice, 4 weeks after implantation. (**B**) Bioluminescence measured from primary tumours on the indicated days post-implantation. Bioluminescence was quantified as indicated on the vertical axes. (**C**) Calculated volume derived from calliper measurement of primary tumours over the same period. (**D**) Bioluminescent images of metastases in control and phenytoin-treated mice. (**E**) Bioluminescence measured from the indicated anatomical sites. (**F**) Bioluminescence measured ex vivo from liver, lungs, and spleen. Data are means ± standard error of the means (SEMs). Asterisks (*’s) indicate statistically significant differences (cf. corresponding controls). Modified from [84].

Subsequently, an orthotopic xenograft mouse model of BCa using metastatic MDA-MB-231 cells injected into the fourth inguinal mammary fat pad of female immunodeficient mice was used to show the involvement of Nav1.5 in tumour progression in vivo (Figure 5A–C) [11]. Using lentiviral shRNA, it was shown that knock-down of Nav1.5 slowed primary tumour growth, reduced MMP9 expression, increased apoptosis, and inhibited local invasion and metastasis to the liver, lungs, and spleen [11]. In addition, using ex vivo tumour tissue slice patch clamp recording, it was shown that functional Na^+^ currents carried by Nav1.5 were maintained in vivo, supporting the notion that VGSC expression is not an artefact of in vitro cell culture conditions [11].

Some work has also been done on VGSC beta subunits. Stable over-expression of β1, which occurs endogenously at low levels in MDA-MB-231 cells [85] as well as in BCa and PCa biopsy tissues [86,87], significantly reduced apoptosis within the primary tumour and increased angiogenesis, tumour growth, and distant metastasis [87]. In contrast, over-expression of β2 in PCa (LNCaP) cells reduced tumour growth when the cells were subcutaneously implanted into immunodeficient mice [88]. In human cancer (including CRCa and NSCLC) cell lines, expression of *SCN3B* (the gene encoding β3) was found to be upregulated by DNA-damaging agents and by overexpression of p53 [89]. Importantly, in vivo (in p53(+/+) mice), *SCN3B* was also similarly upregulated following adriamycin treatment. It was concluded that *SCN3B* was involved in a proapoptotic pathway and may serve as a tumour suppressor [89]. In similar fashion, in mice with β4-silenced MDA-MB-231 cell xenografts, tumour growth, invasiveness, and metastasis were enhanced. These occurred independently of VGSC activity and suggested that *SCN4B* (the gene encoding β4) functions by itself as a tumour suppressor [90]. Consistent with these in vivo data, β4 expression was low/absent in BCa and cervical cancer tissues [90,91]. In addition, in papillary thyroid cancer *SCN4B* was downregulated at both RNA and protein levels in comparison with normal thyroid tissues, and sustained *SCN4B* expression was an independent indicator of favourable recurrence-free survival [92]. Together, these studies suggested that both Nav1.5 and β1 can promote, whereas β2, β3 and β4 may suppress cancer progression in vivo.

**Figure 6:**
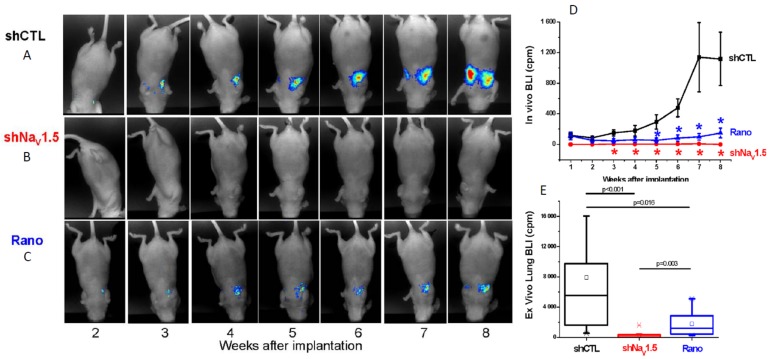
In vivo evidence showing suppression of breast cancer (BCa) metastasis in a mouse (‘tail vein’) model by silencing of Nav1.5 and ranolazine. (**A**) Bioluminescent imaging (BLI) of living nude mice inoculated with human BCa MDA-MB-231 cells and followed over 8 weeks (“shCTL”). Representative images are shown. (**B**) Cells with Nav1.5 ‘silenced’ with short-hairpin (sh) Nav1.5 (“shNav1.5”). (**C**) As for the control, but mice were treated (5 days/week) with 50 mg/kg ranolazine (“Rano”). (**D**) Data quantified from (**A**–**C**). Tumourigenesis in intact mice was quantified as BLI value expressed in “counts per minute” (cpm). Asterisks (*’s) indicate statistically significant differences (cf. shCTL). (**E**) Quantification and statistical analyses of BLI values measured ex vivo from lungs (excised after 8 weeks of experimentation). Modified from [12].

Following on from these observations, several studies have explored the potential value of pharmacologically inhibiting VGSCs in vivo using clinically-approved blockers [93]. Using the same orthotopic xenograft BCa model described above, it was shown that the Class 1b antiarrhythmic and antiepileptic phenytoin (daily intraperitoneal injection 60 mg/kg) significantly reduced proliferation, primary tumour growth, local invasion, and distant metastasis (Figure 5D–F) [84]. In an experimental metastasis model, in which MDA-MB-231 cells are injected intravenously to form lung metastases, it was shown (i) that silencing Nav1.5 completely suppressed metastasis and (ii) that the anti-anginal VGSC-I_NaP_ inhibitor ranolazine (intraperitoneal 50 mg/kg/day) significantly reduced lung colonisation (Figure 6A–E) [12]. In a peritoneal carcinomatosis model, direct administration of the VGSC-inhibiting local anaesthetic lidocaine (100 mg/kg once weekly) following intraperitoneal injection of MDA-MB-231 cells into female immunodeficient mice, resulted in a significant reduction in tumour burden and prolongation of survival [94]. In the 4T1 mouse orthotopic allograft model of BCa, lidocaine (intravenous 1.5 mg/kg bolus followed by 25 min infusion 2 mg/kg/h) administered during tumour resection significantly reduced pulmonary metastasis, but only when combined with sevoflurane and not with ketamine, suggesting that perioperative administration of VGSC inhibitors may have therapeutic value that is dependent on the anaesthetic combinations used [95]. In the same model, also under sevoflurane anaesthesia, both perioperative propofol (5 mg/kg) and lidocaine were shown to lead to a reduction in pulmonary metastases [96]. Lidocaine also enhanced the anti-metastatic effect of cisplatin [97]. In a similar study on hepatocellular carcinoma, lidocaine both suppressed tumour development of human HepG2 cell xenografts in BALB/c nude mice and enhanced sensitivity to cisplatin [98]. Procaine was also found to reduce tumour volume in BALB/c nude mice HLE hepatoma cell xenografts in vivo [99]. The functional role of Nav1.7 in gastric cancer growth was confirmed in a xenograft experiment in vivo [23]. Finally, in the Copenhagen rat model of prostate cancer, ranolazine (2.5–5 µM; oral gavage) decreased lung metastatic burden following subcutaneous implantation of Mat-LyLu cells, without affecting primary tumour growth [50]. 

Taken together, these various studies suggested that administration of VGSC-inhibiting drugs may help to slow cancer progression in vivo by suppressing metastasis, and in some models, primary tumour growth. Also, where it could be studied, such treatments prolonged survival.

## 6. Clinical Studies

The combined in vitro and in vivo preclinical evidence suggest that repurposing VGSC-inhibiting medication to the oncology setting may have therapeutic value. Given that such drugs are in widespread clinical use for other indications, several observational studies have investigated potential associations with cancer outcomes [100,101,102]. For example, it has been shown that the risk of recurrence in patients with BCa or PCa who received general anaesthesia and perioperative lidocaine was reduced compared to those who did not receive lidocaine [103,104]. On the other hand, retrospective analysis of PCa incidence using the Taiwan Longitudinal Health Insurance Database 2005 found no association between VGSC blocker use and cancer risk [105]. Another retrospective cohort study using the QResearch database and seeking to investigate the relationship between prescription of VGSC-inhibiting antiepileptic medication and overall survival of breast, colorectal, and prostate cancer patients demonstrated, rather surprisingly, significantly shortened overall survival in cancer patients exposed to VGSC-inhibiting antiepileptic medications [106]. Although the reasons for this negative association are not clear, it is likely that increased mortality in the drug-exposed group may be the result of confounding by indication, thereby masking any potential beneficial effect in the population [107]. Other studies revealed the predicted inverse association between use of VGSC blocking drugs and survival. Retrospective analysis of cancer occurrence using the United States Food and Drug Administration Adverse Event Reporting System and an insurance claims database (containing 3 million cases) demonstrated significant inverse association between use of VGSC-inhibiting antiepileptic medications and diagnoses of colorectal cancer, lung cancer, gastric cancer, and haematological malignancies [108]. Similarly, a case-control study using the General Practice Research Database showed significantly reduced prescription of VGSC-inhibiting tricyclic antidepressants in colorectal cancer (and glioma) patients, suggesting that these medications may also be effective for reducing recurrence of certain cancers [109]. 

VGSC inhibitors may also have an added benefit in combination with existing treatments. An observational study of records from 253 patients with BCa brain metastases who had undergone whole brain radiotherapy (WBRT) showed that the overall survival of those receiving antiepileptic drugs was significantly longer than for patients not receiving such medication, although this association may be due to reduced mortality from better control of seizures caused by WBRT [110]. Administration of anti-epileptic drugs improved overall survival in Her2^+^ BCa patients and those undergoing surgery [110]. Suppressing the activation of VGSC-I_NaP_ by certain chemotherapeutic drugs, including anthracyclines, could reduce the cardiotoxicity associated with standard-of-care therapies [111]. Such a possibility is the focus of a proof-of-concept clinical study of the efficacy and safety of ranolazine in cancer patients [112]. It has also been shown that epilepsy-related mutations in *SCN1A* (encoding for Nav1.1) are associated with significantly shortened recurrence-free survival in CRCa patients treated with 5-fluoruracil adjuvant chemotherapy [113]. These studies add further evidence supporting the notion that VGSC expression may be useful predictive biomarkers and therapeutic targets, including potentially in combination with other therapies.

On balance, several retrospective observational studies have suggested a positive association between exposure to VGSC-inhibiting medications and cancer outcomes, although the data are partially conflicting. Such studies suffer by design from multiple confounding variables limiting their value. Therefore, there is a compelling case to justify the implementation of appropriately controlled prospective clinical trials. Notably, several trials of VGSC-inhibiting antiepileptic and local anaesthetic drugs are already registered/recruiting. Importantly, within the context the VGSC hypothesis, only one so far is specifically designed to assess the in vivo ability of local anaesthetic agents (e.g., lignocaine) for operable BCa in order to decrease the dissemination of cancer cells during surgery and improve disease-free survival (ClinicalTrials.gov identifier: NCT01916317; estimated completion date: December 2023).

## 7. Conclusions and Future Perspective

In overall conclusion, there is increasing substantial evidence for VGSC expression in tumours in vivo, including in human tissues, at a hierarchy of levels from cellular mRNA to clinical studies. This VGSC expression is functional, whereby channel activity promotes metastatic cell behaviours in vitro and metastasis in vivo. Such expression offers several advantages as an anti-cancer target with significant clinical advantages and possibilities of application, as summarised in Table 2. *Diagnostics*: The early expression of the VGSC in the cancer/metastatic process would make it an ideal biomarker in decision-making, especially with regards to possible radical surgery. Furthermore, the possible cancer specificity of VGSC subtypes such as nNav1.5 (and an antibody for it) can facilitate this process, as well as help identify those patients who might benefit from VGSC-based therapies. *Therapeutics*: We propose that it would be timely to test VGSC blockers as anti-metastatic drugs in carefully planned clinical trials. An obvious first step would be to test existing clinical drugs, such as anti-epileptics (e.g., phenytoin) or anti-angina drugs (e.g., ranolazine), on the basis of ‘repurposing’ [36,114,115].

## Figures and Tables

**Table 1:** Voltage-gated sodium channel (VGSC) subtype expression in human carcinomas.

Carcinoma	VGSC Subtype(s)	Comment(s)	Reference(s)
Breast	nNav1.5 (and (n)Nav1.7)	Dominant at mRNA level and functional contribution to invasiveness/metastasis verified in vitro and in vivo	[9,10,11,12]
Colon	nNav1.5	Dominant at mRNA level and expressed early in invasiveness; functional contribution to invasiveness verified in vitro and in vivo	[13,14,15]
Prostate	(n)Nav1.7 (and Nav1.6)	Dominant at mRNA level; specific functional contribution to invasiveness tested using peptide toxins	[8,16,17,18]
Non-small cell lung carcinoma (NSCLC)	Nav1.7	Dominant at mRNA level; potentiation of invasiveness demonstrated by use of siRNA	[19]
Mesothelioma	Nav1.2, Nav1.6, and Nav1.7	VGSC activity shown to promote migration in vitro, but the subtype(s) responsible not determined	[20]
Cervical	(n)Nav1.6	Dominant at mRNA level; over-expression potentiated invasiveness	[21,22]
Stomach	Nav1.7	Dominant at mRNA level; silencing suppressed tumour growth in mouse model in vivo	[23]
Ovary	Nav1.5	Dominant at mRNA level; E3 antibody suppressed in vivo growth and in vitro invasiveness	[24,25]
Melanoma	Nav1.5	Expression induced membrane potential depolarization and inhibited Ca^2+^ uptake	[26]
Oral squamous cell carcinoma	Nav1.5	siRNA confirmed in vitro potentiation of proliferation and invasiveness	[27]
Astrocytoma	nNav1.5	siRNA confirmed in vitro potentiation of proliferation and invasiveness	[28]
Neuroblastoma	nNav1.5	Where ‘neonatal’ Nav1.5 splicing was first described, but role in cancer not investigated	[29]
Endometrium	Nav1.7	Channel block attenuated in vitro cell invasion; expression was associated (i) positively with tumour size and local lymph node metastasis, and (ii) negatively with survival (5–10 years)	[30]

Although different carcinomas express different subtype(s) of VGSC, two appear most common—Nav1.5 and Nav1.7. Where studied, the channels were found to occur in ‘neonatal’ splice form (indicated by the prefix “n”). Although brain tumours are not covered in this review, astrocytoma and neuroblastoma are included for completeness. In most cases, the role of VGSC activity in promoting invasiveness was tested using tetrodotoxin (TTX). In addition, some studies also demonstrated the role of the ‘dominant’ VGSC subtype by siRNA or antibody. Only exemplary references are given.

**Table 2:** Essential properties of the VGSC that would make it a clinically viable anti-metastatic target.

VGSC Property	Clinical Consequence(s)	Reference(s)
Expression much higher in strongly vs. weakly metastatic cancers (by up to several orders of magnitude at mRNA level)	Potential functional diagnostic molecular biomarker	e.g., [9,16,40]
Expression is early and upstream of the genes driving invasiveness	Potential functional ‘early’ marker, ideal for diagnostics	[14]
Upregulation maintained at protein and functional (signalling) levels	Diagnostics can be extended to conventional immunohistochemistry and even clinical imaging of tissue sodium possibly resulting from channel activity (e.g., ^23^Na-MRI); expression can be used to determine treatment strategy and efficacy	[9,16,57,65,66]
Gene/protein expressed in neonatal splice form in several carcinomas; targetable by antibody	Diagnostics can be made even more specific; antibody can also be used as a drug (i) to block channel activity/metastasis and/or (ii) kill tumour cells (in antibody-drug conjugate mode)	[9,10,56]
Activity-dependent regulation (positive feedback)—VGSC blockers suppress both channel activity and expression	VGSC blockage would suppress both activity and expression of the channel, thereby providing long-term benefit to cancer patients taking VGSC drugs	[60,116]
Promotes a range of cellular behaviours integral to the metastatic cascade in vitro, as well as metastasis per se in vivo	Therapeutic potential—possible ‘repurposing’ of existing VGSC drugs, I_NaP_ blockers (as well as novel antibody), possibly with minimal side effects	[11,12,36,114]
Functional expression under the control of steroid hormones and growth factors	Therapeutic potential extended to ‘combination’ treatments	[19,61]

VGSC, especially nNav1.5, expression in carcinomas has several independent properties that would make an attractive clinical target for both diagnostics and therapeutics. Only exemplary references are given.
